# Impact of Modified Diet, Swallowing Exercises, and Electrostimulation on Quality of Life of Older Patients Suffering from Oropharyngeal Dysphagia

**DOI:** 10.3390/medicina60071021

**Published:** 2024-06-21

**Authors:** Margarita Rugaitienė, Vita Lesauskaitė, Ingrida Ulozienė, Lukas Smičius, Gytė Damulevičienė

**Affiliations:** 1 Clinical Department of Geriatrics, Lithuanian University of Health Sciences, 44307 Kaunas, Lithuaniagyte.damuleviciene@lsmu.lt (G.D.); 2 Department of Otorhinolaryngology, Lithuanian University of Health Sciences, 44307 Kaunas, Lithuania; 3 Department of Infectious Diseases, Lithuanian University of Health Sciences, 44307 Kaunas, Lithuania

**Keywords:** oropharyngeal dysphagia, dysphagia screening, neurostimulation, modified diet, fiberoptic endoscopic evaluation of swallowing, quality of life

## Abstract

*Background and Objectives*: Oropharyngeal dysphagia is defined as a swallowing disorder in which it becomes difficult to form a bolus and move food from the mouth to the proximal part of the esophagus. Several factors can cause this disorder in geriatric patients. With oropharyngeal dysphagia, the patient’s social isolation and the risk of depression increase, while the quality of life deteriorates. *Materials and Methods*: In this study, oropharyngeal dysphagia was suspected based on the EAT-10 questionnaire and diagnosed with the water drink test and endoscopic swallowing evaluation, which assesses the aspiration risk by using an eight-point Penetration–Aspiration scale. Patients with oropharyngeal dysphagia received complex treatment: exercises to strengthen the swallowing muscles, electrostimulation of the swallowing muscles, and a modified diet. The quality of life of 64 patients was assessed by using the DHI, SWAL-QoL, and EAT-10 questionnaires before complex treatment and after treatment. The results show that the quality of life improved after the complex treatment of oropharyngeal dysphagia. *Results*: The mean age of patients was 77.8 (9.1) years, and 56.3% of patients were women. At baseline, mild oropharyngeal dysphagia was found in 18.8% of patients; moderate—in 51.6%; and severe—in 29.7%. Aspiration risk was low in 28.1% of patients; medium—in 39.1%; and high—in 32.8%. The severity of oropharyngeal dysphagia and aspiration risk significantly decreased after treatment (*p* = 0.002). The EAT-10 score mean was 15.23 (8.92) points before treatment and decreased to 11.50 (6.12) points after treatment (*p* < 0.001). Before treatment, the DHI physical score was 15.75 (6.813), the DHI functional score was 14.56 (8.659), and the DHI emotional score was 11.06 (7.848) (*p* < 0.001), and after complex treatment, the DHI physical score was 14.56 (8.659), the DHI functional score was 9.74 (7.165), and the DHI emotional score was 7.94 (6.588) (*p* < 0.001). The total SWAL-QoL score mean was 132.71 (34.392) points before treatment and increased to 152.42 (30.547) points after treatment (*p* < 0.001). *Conclusions*: Complex treatment of oropharyngeal dysphagia plays an important role in improving the quality of life and reducing aspiration risk in older people affected by this condition.

## 1. Introduction

Oropharyngeal dysphagia (OD) is one of the leading causes of death in both children and adults with neurological disorders, so early diagnosis and treatment of OD are crucial [1]. This syndrome can occur in all stages of life, and its prevalence in the general population varies between 2.3% and 16% [2]. However, the prevalence of OD as a geriatric syndrome is higher among the elderly: 30% to 40% among the elderly living independently, 44% among those admitted to geriatric intensive care, and 60% among hospitalized elderly patients [3]. Due to age-related changes in swallowing physiology and factors such as disease, OD is more common in people over the age of 65 and patients with neurological conditions such as stroke, multiple sclerosis, and Parkinson’s disease [4]. One of the most common causes of OD is stroke. Approximately 13.7 million people worldwide experience stroke each year, and almost half of these people experience varying degrees of functional impairment that seriously affects their quality of life. OD is a prevalent disorder after stroke: it occurs in about 80% of cases [5]. OD can lead to dehydration, malnutrition, and aspiration, which can lead to respiratory infections and pneumonia. Mortality in patients due to aspiration pneumonia is up to 50%. The leading causes of one-year mortality in stroke survivors are respiratory infections and aspiration pneumonia, two well-known complications of OD after stroke [6]. To determine the goal of treatment and to select the best-suited treatment method for the underlying disease, it is important to diagnose the underlying pathology causing OD, which requires a multidisciplinary approach. OD treatment aims to improve swallowing safety by changing food texture, fluid consistency, and/or feeding posture. Studies show that the volume and viscosity of the bolus alters the biomechanics of swallowing. Changing the texture of the bolus can reduce the risk of irregular bolus movement during swallowing. Increasing bolus viscosity may reduce the risk of aspiration, but such changes in bolus physical properties are also associated with altered palatability and increased pharyngeal residual volume (stasis) [7]. Another part of complex OD treatment is neuromuscular electrical stimulation of swallowing muscles. During the procedure, the swallowing muscles are stimulated with short electrical impulses. This method is widely used in the treatment of OD patients. Electrical stimulation strengthens the muscles involved in swallowing and facilitates reflex swallowing through sensory stimulation [8]. Physical exercises aimed at strengthening the swallowing muscles are also included in the complex treatment of OD. The results of many clinical studies have been associated with positive changes in swallowing physiology, including improvements in swallowing initiation, improvement in laryngeal elevation, and reduction in post-swallow stasis [9]. Most patients find that OD harms their lives: it reduces self-confidence and affects their social life, work, and leisure [10].

There are about 20 health-related quality-of-life and functional health status questionnaires for patients with oropharyngeal dysphagia. When analyzing research on health-related quality-of-life and functional health status questionnaires, it was found that the Dysphagia Handicap Index (DHI) and Swallowing Quality of Life (SWAL-QoL/SWAL-CARE) meet the methodological requirements best and are mostly recommended [11]. That is why these questionnaires were chosen for this study.

The aim of this study was to diagnose OD and evaluate aspiration risk (AR) and the quality of life of older patients before and after complex treatment.

## 2. Materials and Methods

***Subjects:*** A total of 64 geriatric patients with oropharyngeal dysphagia due to various causes were included in this study. Patients treated in the Department of Geriatrics and Department of Physical Medicine and Rehabilitation No. 2 of LSMU Kaunas Hospital from May 2021 to April 2023 were included in the study. Inclusion criteria: patients 60 years old and older, suspected swallowing disorder during hospitalization—oropharyngeal dysphagia—and the patient’s written consent to participate in the study and ability to speak and understand Lithuanian. Exclusion criteria were marked respiratory or heart failure: shortness of breath (respiratory rate > 20 bpm and SpO_2_ < 90%), tachycardia (HR > 100 bpm), very severe malnutrition (pronounced exhaustion and marked signs of sarcopenia), final stage of oncological disease, or severe dementia. The study protocol was approved on 23 October 2020 by The Kaunas Regional Biomedical Research Ethics Committee, permit No. BE-2-12.

***Research methods:*** Patients suspected of oropharyngeal dysphagia were subjected to a water swallow test according to the methodology approved by LSMU Kaunas Hospital. Water swallow test method: The patient’s speech is evaluated (voice quality), and saturation is measured (by using a pulse oximeter); the patient, sitting at an angle of about 90°, in a chin-down position, is given 1 teaspoon (5 mL) of water to drink. Possible signs of aspiration observed: cyanosis of the face, altered voice, and decreased saturation (≥3%). If no signs of OD are observed, the amount of water is increased to 15–20 mL (1 tablespoon) of water. In the absence of signs of OD, the patient is instructed to drink 50 (up to 100 mL) of water, which the patient drinks from a cup independently, or the cup is held by the researcher. In the presence of OD symptoms, the sample is evaluated as positive, and OD is determined.

In subjects who met the criteria, the severity of dysphagia and aspiration risk were assessed endoscopically. An endoscopic swallowing examination was performed by using a 3.7 mm HD Video Rhino-Pharyngo-Laryngoscope (STORZ, Tuttlingen, Germany).

Fiberoptic endoscopic evaluation of swallowing (FEES) consists of three parts: (1) evaluation of mouth and throat anatomy, salivary gland secretion, tongue retraction, pharyngeal wall movements, vocal cord mobility, and epiglottis closure; (2) the patient is given a certain amount and a certain consistency of liquid and solid food to swallow, and during this process, the safety and efficiency of swallowing is assessed; (3) preparation of bolus for swallowing, formation of a bite (bolus), chewing, driving force of the tongue and swallowing initiation, and clearing of the pharynx are evaluated. The following are also assessed: bolus retention in the pharynx; aspiration before, during, or after swallowing; and bolus return from the esophagus. FEES was performed for each patient twice: before and after complex treatment.

During the study, the patients were given 3, 5 mL, and 10 mL of water to swallow (after assessing the patient’s safety), followed by 10 and 15 mL of a thick drink like nectar, then 10 and 15 mL of a thick drink like honey, then 10 and 15 mL of pudding, and in the last stage, 20 g of a cracker (Table 1). Drinks and food were colored with food coloring. In the case of very high suspicion of OD, the study was started with the nectar-like thick drink, and after assessing the high risk of aspiration during the study, solid food was not used.

Aspiration risk was evaluated by using the Penetration–Aspiration scale (Rosenbek, 1996) [12,13]: 1 point—no penetration nor aspiration; 2–5 points—penetration; 6–8 points—aspiration. Penetration refers to the bolus moving to the pharynx and coming into contact with the vocal folds but not going beyond them, while aspiration is the entry of the bolus (all or part of it) beyond the vocal folds into the trachea [13].

Oropharyngeal dysphagia was classified as mild (mild dysfunction during the oral or pharyngeal phase that is corrected by dietary modification), moderate (dysfunction and signs of aspiration are evident but can be corrected by dietary modification), or severe (significant dysfunction and clear aspiration, and artificial nutrition is required). Aspiration risk was evaluated by using the Penetration–Aspiration scale and was classified as low (P-A scale 1–3 points), medium (P-A scale 4–5 points), or high (P-A scale 6–8 points).

The patients’ quality of life was evaluated before and after complex treatment of oropharyngeal dysphagia by using three questionnaires: Dysphagia Handicap Index (DHI), Swallowing Quality of Life (SWAL-QoL/SWAL-CARE), and Eating Assessment Tool (EAT-10). The questionnaires DHI and SWAL-QoL/SWAL-CARE were translated into Lithuanian after obtaining the consent of the authors of the questionnaires.

The DHI questionnaire consists of 25 statements, which are separated into three categories: statements assessing the patient’s functional status (9 statements; the letter *F* is added to the statement number in the questionnaire), statements assessing the physical aspects of the swallowing disorder (9 statements; the letter *P* is added to the statement number in the questionnaire), and statements assessing the patient’s emotional state and quality of life (7 statements; the letter *E* is added to the statement number in the questionnaire). Possible answers to the questions in the questionnaire: never, sometimes, and always. Assessment: never—0 points; sometimes—2 points; and always—4 points. The maximum score is 100. The more points the patient scores, the more severe the OD. If the patient scores 0 points, it is considered that the patient does not feel/has no difficulty swallowing. The patient rates the severity of dysphagia on a scale from 1 (normal) to 7 (severe dysphagia) [10]. As scores decrease, quality of life improves. The time necessary for filling out the questionnaire is about 20 min.

The SWAL-QoL and SWAL-CARE questionnaire consists of two parts. The first part consists of 9 scales (44 items in total), with which the burden caused by the swallowing disorder, the duration of eating, the desire to eat, food choice, fear, mental health, social functioning, communication, sleep, and fatigue are assessed. There is also a symptom-frequency scale where each item is rated from 1 to 5 (1 for worst quality of life and 5 for best quality of life). The second part of the questionnaire (15 questions) helps to evaluate the effectiveness of the prescribed dysphagia treatment. The patient chooses the most appropriate answer to the statements on a five-point Likert scale, which are then converted into a score from 0 to 100. The lower the score, the worse the quality of life [14]. The time necessary for filling out the questionnaire is about 20 min.

The Eating Assessment Tool, EAT-10, was developed by author Belafsky [15] and consists of 10 questions (the original English questionnaire comprises 235 questions). This 10-question questionnaire helps to assess dysphagia, its severity, and at the same time, the patient’s quality of life. Each question is evaluated from 0 to 4 points. The higher the score, the more pronounced the swallowing disorder. The lower the score, the better the quality of life. If more than 3 points are collected, an examination for dysphagia is required. This questionnaire has been translated into Lithuanian and approved by the LSMU Department of Geriatrics; thus, it can be used freely. The time necessary for filling out the questionnaire is up to 5 min.

***Treatment***: The complex treatment of oropharyngeal dysphagia consisted of transcutaneous electrical stimulation of the swallowing muscles, diet modification, and exercises for strengthening the swallowing muscles.

Transcutaneous electrical stimulation is a dysphagia treatment method in which mild electrical impulses stimulate the muscles in the neck area involved in swallowing. An electric impulse is emitted through an electrode attached to the skin, which is fixed in the projection of the muscle to be affected. The emitted electrical impulse affects the peripheral nerve, spreads through it, and after reaching the muscle innervated by this nerve, causes its contraction [8]. The strength of the electric impulse was selected individually, according to the degree of tolerance of the patient. During the study, this procedure was performed on the patient by certified healthcare professionals (researcher or physiotherapists). The duration of the procedure was 30 to 40 min. The procedure was performed with a VitalStim device (ChattaNooga, Guildford, UK), with a median impulse intensity of 7 mA.

Diet modification was assigned to each patient individually based on the severity of oropharyngeal dysphagia and risk of aspiration as determined by the endoscopic swallowing assessment study. Patients with mild OD were given the therapeutic diet MM5 Minced and Moist, and those with moderate or severe OD were given the therapeutic diet PU4 Pureed (Table 2).

Each patient was trained in 7 exercises to strengthen the swallowing muscles: effortful swallow; tongue-hold swallow; supraglottic swallow; shaker exercise; Mendelsohn maneuver; effortful pitch glide; chin tuck. The patients were taught the exercises by a physiotherapist, and each patient trained with the physiotherapist for about 20–30 min every day.

## 3. Observation

1. Clinical effectiveness: Reduction in oropharyngeal dysphagia symptoms and improvement in quality of life were evaluated by using the DHI questionnaire, EAT-10 questionnaire, and SWAL-QoL and SWAL-CARE questionnaires.
2. Swallowing function: The clinical effect of treatment was assessed by endoscopic evaluation of swallowing before and after complex treatment.

In the study, two patients after 7 treatment sessions were excluded from the study due to developed pneumonia, respiratory failure, and later death, so their results were not included in the calculation of correlations, change in risk of aspiration, and change in OD.

***Statistical analysis*** was conducted by using SPSS 28.0 software (IBM Corp., released 2021; IBM SPSS Statistics for Windows (version 22H2); Version 28.0; Armonk, NY, USA). In the descriptive analysis, for continuous indicators, the means ± standard deviation (SD) were calculated, and for categorical variables, absolute prevalence (*n*) and percentages (%). In inferential analysis, the relationships between categorical variables were assessed by using the chi-squared test with Z-values, and the associations among continuous variables, by using the Pearson correlation coefficient. The level of statistical significance was set at *p* < 0.05.

## 4. Results

The mean age of the patients was 77.8 (9.1) years, and 56.3% of patients were women. The average duration of patient hospitalization was 11.7 days. The PA scale score median was 4 points (IQR 3–6) before treatment and significantly decreased to 3 points (IQR 2–4) after treatment (*p* < 0.001).

**Figure 1:**
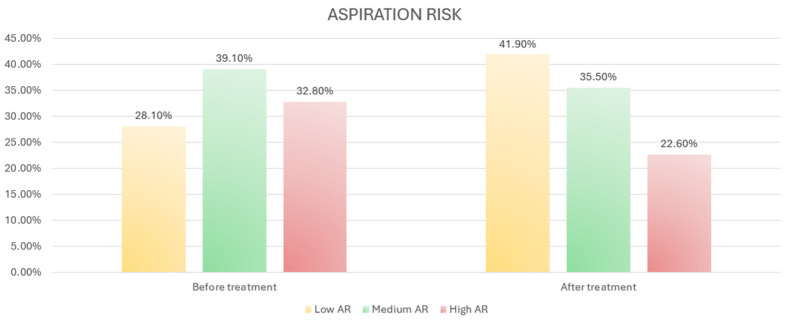
Percentage change in aspiration risk after treatment.

Aspiration risk (AR) significantly decreased after treatment (*p* = 0.002) (Figure 1).

**Figure 2:**
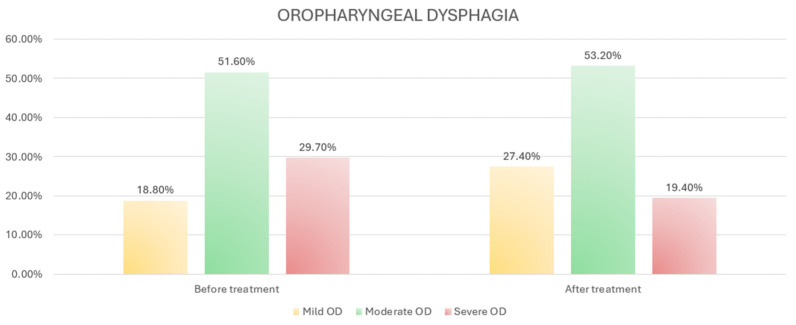
Percentage change in severity of oropharyngeal dysphagia after treatment.

The severity of oropharyngeal dysphagia significantly decreased after treatment (*p* = 0.002) (Figure 2).

The EAT-10 score mean was 15.23 (8.92) points before treatment and decreased to 11.50 (6.12) points after treatment (*p* < 0.001). The EAT-10 score > 20 was observed in 23.4% of patients before treatment and decreased to 6.5% after complex treatment (*p* = 0.002).

## 5. Reliability of Questionnaires Used

The internal consistency of the EAT-10 questionnaire was good, with Cronbach’s α ranging from 0.89 to 0.79. The internal consistency of the DHI questionnaire was also good, with Cronbach’s α ranging from 0.93 to 0.94, with the lowest estimate for the physical subscale. The internal consistency of the SWAL-QoL and SWAL-CARE questionnaire was very good, with Cronbach’s α of 0.97 (Table 3).

Changes in all quality-of-life questionnaire scores after treatment were significant, with quality of life being improved (Table 4).

The DHI has additional question about respondents’ OD intensity, which is evaluated from 1 to 7 points (the higher the score, the more severe the OD). Before treatment, the median score was 5 points (28.1%) (moderate severity), and after treatment, 4 points (48.4%) (moderate severity).

## 6. Correlations

Aspiration risk was positively correlated with the physical subscale before and after treatment and with the functional and emotional subscales of the DHI only before treatment and not correlated after treatment. Aspiration risk was not correlated with the emotional and functional subscales after treatment, as OD treatment time during hospitalization was not long enough to change old habits and form new ones. Improvement in OD was the most noticeable in the physical subscale, as the questions in this subscale are directly related to swallowing.

Aspiration risk was positively correlated with EAT-10 before treatment but not after treatment (Table 5).

Aspiration risk was positively correlated with general statements, physical problems, and communication before and after treatment, while it was correlated with eating aspects, effects on diet and habits, and emotional well-being only before treatment. Aspiration risk was not correlated with SWAL-CARE before or after treatment (Table 6).

DHI was positively correlated with EAT-10. As the number of DHI scores increased, the quality of life worsened; similarly, as the number of EAT-10 scores, the quality of life worsened (Table 7).

Before treatment, EAT-10 was positively correlated with all parts of the DHI (as the EAT-10 score increased, so did the DHI score) and negatively correlated with SWAL-QoL parts 1–9 and the total score (as the EAT-10 score increased, the SWAL-QoL score decreased). There was no correlation between SWAL-CARE and EAT-10.

After treatment, EAT-10 was positively correlated with all DHI parts, all parts and the total score of SWAL-QoL, and SWAL-CARE (Table 8).

The DHI was negatively correlated with SWAL-QoL both before and after complex treatment of oropharyngeal dysphagia. As the DHI score increased, the quality of life worsened, and as the SWAL-QoL score decreased, and the quality of life also worsened. SWAL-CARE did not correlate with any part of the DHI (Table 9).

## 7. Discussion

In old age, oropharyngeal dysphagia can be caused by various factors: stroke, dementia, neurodegenerative diseases (Parkinson’s disease, motor neuron disease (lateral amyotrophic sclerosis), myasthenia gravis, and multiple sclerosis), and dry mouth (xerostomia) [14]. In our study, oropharyngeal dysphagia was mostly caused by ischemic stroke. Other causes—Parkinson’s disease, vascular dementia, or lateral amyotrophic sclerosis—were isolated. In some individuals, the cause of OD remains unknown. This geriatric syndrome is associated with many complications—malnutrition, dehydration, and aspiration pneumonia. As a result of these complications, the number of hospitalizations and patient mortality increase [16]. Of the patients in this study, four patients died of aspiration pneumonia. Two patients died during the study period (after seven treatment sessions) due to aspiration pneumonia, the other two died 6 months after the end of the study.

To prevent deterioration of patients’ quality of life and life-threatening complications, questionnaires are used to help identify patients experiencing swallowing disorders or complications caused by them [17]. A study conducted by Timmerman and colleagues [18] revealed that the questionnaire Swallowing Quality of Life (SWAL-QoL/SWAL-CARE) best assesses the psychometric parameters of patients, while the questionnaire Dysphagia Handicap Index (DHI) is the easiest to apply [19]. Therefore, these two questionnaires and the EAT-10 questionnaire were chosen to assess the quality of life of the participants in this study. Other questionnaires used for the diagnosis of dysphagia are more focused on the specific etiology of dysphagia and therefore are only suitable for a limited number of patients. This are the Brief Esophageal Dysphagia Questionnaire (BEDQ [20]), Dysphagia in Multiple Sclerosis (DYMUS) [21], the Munich Dysphagia Test—Parkinson’s disease (MDT-PD) [22], and many others. Other questionnaires can be aimed at a specific age group of patients: children, adults, or older patients.

The DHI is widely used in both research and clinical practice and is reliable, which is why it was chosen as one of the main questionnaires. The DHI questionnaire is considered understandable by patients and easy to apply, as it does not require the assistance of a clinician during completion. It is important to emphasize that the DHI questionnaire allows the patient to self-assess the severity of perceived dysphagia on a scale from 1 (normal) to 7 (severe dysphagia), so it is possible to objectively assess the patient’s condition or the effectiveness of the prescribed dysphagia treatment. However, for the assessment to be accurate, it is important to have well-defined norms for interpreting the results. Five studies were included in the analysis, and the results showed a wide range of DHI scores, from 27.33 to 77.72. Such a wide range can be attributed to various other factors, such as culture, environment, severity of dysphagia, and cause of OD [10]. The patients in our study were diagnosed with OD for various reasons (neurological disorders, dementia, and unknown reasons), and the scores of the DHI questionnaire were different in each part, but after the complex treatment of OD, these scores were statistically significantly reduced, indicating an improvement in the quality of life. The DHI, as an important measure of quality of life, was also used by Hashemian et al. in a study that evaluated OD before and after thyroidectomy. The study observed a statistically significant decrease in questionnaire scores (*p* < 0.001) and an improvement in quality of life immediately after OD treatment and after 6 weeks (*p* < 0.001) [23].

Currently, this questionnaire has been successfully translated and adapted into Polish [24], Hebrew [25], Persian [26], Korean [27], and other languages. During this study, this questionnaire was translated into Lithuanian. Silbergleit and colleagues’ reliability and quality assessment of the DHI questionnaire [28] found that the overall internal consistency of the final version of the questionnaire was good (Cronbach’s α 0.94), and the reliability of the questionnaire in terms of repeated tests for the overall result, as well as for individual groups of questions, was strong (Pearson correlation coefficient of 0.75–0.86), which shows that the DHI is a reliable tool for assessing the severity of perceived dysphagia symptoms and the impact on quality of life [10]. The internal consistency of the questionnaires translated into Lithuanian was the same—0.94 (good).

Another important questionnaire widely used in clinical practice to assess quality of life is Swallowing Quality of Life (SWAL-QoL/SWAL-CARE). This questionnaire was developed by McHorney and colleagues in 2000 to provide a reliable tool for assessing quality of life and treatment effectiveness in patients with swallowing disorders [14]. This questionnaire is completed before video fluoroscopy or endoscopic swallowing evaluation. The questionnaire consists of two parts: SWAL-QoL and SWAL-CARE.

The patients in our study easily understood the questionnaire questions and answered them fluently before and after complex OD treatment. In our study, before complex treatment, the majority of patients had moderate OD (51.6%; *n* = 33). The patient filled out this questionnaire based on their well-being in the last month [14,27]. This questionnaire has also been translated into other foreign languages. SWAL-QoL is suitable for patients with dysphagia of various etiologies, for instance, those related to oncological, vascular, and neurological diseases; trauma; and other chronic conditions [29]. Some authors emphasize the complex wording of the questions of this questionnaire, compared with the questions of the DHI questionnaire, and the fact that several answer options are possible for the statements [10]. Nevertheless, SWAL-QoL remains a reliable tool both for dysphagia screening and for assessing the severity of the dysphagia experienced by measuring the patient’s tolerance of foods with different textures and consistencies [30]. The evaluation of the questionnaire by the questionnaire authors found that the reliability of five questionnaire scales ranged from 0.80 to 0.89 (the scale was considered reliable when Cronbach α ≥ 0.80) and that the internal consistency coefficient of the other seven scales exceeded 0.90. When assessing reliability in terms of repeated tests for the overall result, the median Pearson correlation coefficient was 0.76, which shows that the scales of this questionnaire have excellent internal consistency, reliability, and short-term stability. It has also been shown that the severity of dysphagia, assessed by the texture of the food consumed, correlates with poorer quality of life [14].

The risk of aspiration before treatment was negatively correlated with SWAL-QoL parts 1–8 and the total score; however, it did not correlate with part 9 before and after treatment, where the questionnaire’s responses are not specific to dysphagia but refer to more general symptoms that may occur frequently in older patients with various chronic conditions. Following treatment, aspiration risk did not correlate with parts 2, 4, 6, and 8. These parts focus on the patient’s eating habits and the psychological aspects of OD and living in the community, all of which require more time than the duration of hospitalization to allow change to happen. SWAL-CARE did not correlate with the risk of aspiration either before or after treatment. According to a study conducted in Iran, the application of swallowing muscle strengthening exercises and a modified diet in 72 patients with OD of varying severity resulted in a statistically significant improvement in quality of life and a marked increase in SWAL-QoL total score from 117.63 (26.37) to 151.63 (28.21) [31]. The answers to the SWAL-CARE questionnaire are not related to OD symptoms or the patient’s well-being, as in this questionnaire, the patient evaluates medical professionals about the information provided about OD and the help in the management of OD. Because the patients had not received appropriate help for OD prior to the survey, low SWAL-CARE scores were obtained. After treatment, these scores increased in our study, suggesting that as the patient’s well-being and OD symptom control improve, the patient’s appreciation of healthcare professionals for the help and additional information provided improves.

Another questionnaire designed to assess the risk and severity of oropharyngeal dysphagia is the Swallowing Screening Tool, EAT-10. This is a questionnaire Belafsky and colleagues developed in 2008 to screen patients at high risk for swallowing disorders. The reliability and sensitivity of the questionnaire were evaluated in a study conducted by Belafsky in 482 patients. The quality of life of the subjects was assessed by this questionnaire, and its score decreased statistically significantly after OD treatment. Belafsky and colleagues, after examining the final version of the questionnaire, estimated that the internal consistency of the EAT-10 questionnaire was good, with Cronbach’s α ranging from 0.947 to 0.960. Also, when the internal reliability of repeated tests for the total score was performed, the correlation coefficient ranged from 0.72 to 0.91. This means that EAT-10 is a reliable tool both for dysphagia screening and for evaluating treatment effectiveness [15]. This questionnaire consists of 10 questions that the patients answer themselves. It is observed that scores greater than 15 increase the risk of aspiration in patients by 2.2 times [32]. EAT-10 is a world-renowned screening questionnaire and has been successfully translated into Spanish [33], French [34], and other languages. It is worth emphasizing that EAT-10 is also not limited to one etiology of dysphagia and can be used for both oropharyngeal and esophageal dysphagia [15]. The EAT-10 questionnaire is reliable and has been used for a long time in various studies around the world [35].

The internal consistency of the EAT-10 questionnaire in this study was also good, with Cronbach’s α ranging from 0.89 to 0.97. The positive effect of the complex OD treatment is also reflected in the evaluation of the average scores of the EAT-10 questionnaire, which decreased from 15.23 before the treatment to 11.1 after complex OD treatment. Aspiration risk was positively correlated with EAT-10 only before treatment. However, the questions in EAT-10 are more focused on the patient’s lifestyle while living in the community, so for this reason, hospitalized patients did not experience any changes in this area.

## 8. Conclusions

Thus, summarizing the results of the study, it can be said that complex treatment of oropharyngeal dysphagia plays an important role in improving the quality of life and reducing aspiration risk in older people affected by this condition. By applying a multidisciplinary approach to treatment, where the main focus is on rehabilitation, diet modification, and teaching geriatric patients and their relatives, it is possible to achieve positive results not only in physical health and daily functioning but also in emotional well-being and self-esteem increase.

## Figures and Tables

**Table 1:** Levels of drink thickness used in the study.

Level of Thickness *	Water, mL	Thickener Nutilis (*Nutricia*)	Viscosity mPa·s (50 s^−1^)	Texture **
Level 2: mildly thick	200	2 measuring scoops	294.20	Nectar
Level 3: moderately thick	200	3 measuring scoops	960.05	Honey
Level 4: extremely thick	200	5 measuring scoops	2858.70	Pudding

* Flow Test (The International Dysphagia Diet Standardization Initiative Committee (IDDSI)), ** National Dysphagia Diet (NDD). Viscosity was measured by rheometer Anton Paar MCR 92.

**Table 2:** Modified diet used in the study.

Modified Diet *	Characteristics
**Food**	
Level 5 MM5 Minced and Moist (TM in LSMU Kaunas Hospital)	4–5 services 2100 kcal 15% protein 55% carbohydrates 30% fat
Level 4 PU4 Pureed (P3Ptm in LSMU Kaunas Hospital)	4–5 services 1800 kcal 20% protein 50% carbohydrates 30% fat
**Drinks**	
Level 4 Extremely thick	All drinks (water, tea, coffee, and juice) thickened until ~2858.70 mPa·s (50 s^−1^)
Level 3 Moderately thick	All drinks thickened until ~960.05 mPa·s (50 s^−1^)
Level 2 Mildly thick	All drinks thickened until ~294.20 mPa·s (50 s^−1^)

* IDDSI (The International Dysphagia Diet Standardization Initiative Committee).

**Table 3:** Reliability of quality-of-life questionnaires (Cronbach’s alpha).

Scale	Subscale	Number of Questions	Evaluation I	Evaluation II
EAT-10		10	0.89	0.79
DHI	Total	25	0.93	0.94
	Physical	9	0.76	0.79
	Functional	9	0.86	0.85
	Emotional	7	0.87	0.86
SWAL	QoL	44	0.97	0.97
	CARE	15	0.97	0.97

**Table 4:** Changes in the DHI, SWAL-QoL/SWAL-CARE and EAT-10 quality-of-life questionnaire scores after complex treatment of oropharyngeal dysphagia.

Questionnaire	Mean (SD) before	Mean (SD) after	*p*-Value
**DHI**			
Physical	15.75 (6.813)	14.56 (8.659)	<0.001
Functional	14.56 (8.659)	9.74 (7.165)	<0.001
Emotional	11.06 (7.848)	7.94 (6.588)	<0.001
**SWAL-QoL**			
Part 1 (General statements)	5.67 (2.476)	6.52 (2.414)	<0.001
Part 2 (Eating aspects)	14.86 (5.133)	17.16 (4.560)	<0.001
Part 3 (Physical problems)	43.54 (10.781)	50.32 (9.554)	<0.001
Part 4 (Effects on diet and habits)	5.63 (2.119)	6.90 (2.094)	<0.001
Part 5 (Communication)	6.22 (2.229)	7.32 (2.039)	<0.001
Part 6 (Concerns)	12.09 (4.050)	13.94 (3.401)	<0.001
Part 7 (Emotional well-being)	15.30 (5.242)	17.65 (4.886)	<0.001
Part 8 (Social life)	15.36 (5.440)	16.61 (4.823)	<0.001
Part 9 (Physical symptoms)	13.75 (4.205)	16.00 (4.391)	<0.001
Total	132.71 (34.392)	152.42 (30.547)	<0.001
**SWAL-CARE** (Clinician’s assessment)	51.59 (13.302)	56.68 (12.877)	<0.001
**EAT-10**	15.23 (8.921)	11.50 (6.124)	<0.001

**Table 5:** Correlation of quality-of-life questionnaires DHI and EAT-10 with aspiration risk.

Questionnaire	Correlation Coefficient and *p*-Value before Treatment	Correlation Coefficient and *p*-Value after Treatment
DHI	0.33, 0.008	0.26, 0.044
Physical	0.37, 0.003	0.32, 0.013
Functional	0.28, 0.028	0.25, 0.059
Emotional	0.27, 0.035	0.17, 0.185
EAT-10	0.29, 0.020	0.06, 0.630

**Table 6:** Correlation of quality-of-life questionnaire SWAL-QoL/SWAL-CARE with aspiration risk.

SWAL-QoL	Correlation Coefficient and *p*-Value before Treatment	Correlation Coefficient and *p*-Value after Treatment
Part 1 (General statements)	−0.29, 0.022	−0.27, 0.038
Part 2 (Eating aspects)	−0.33, 0.009	−0.20, 0.122
Part 3 (Physical problems)	−0.33, 0.009	−0.31, 0.015
Part 4 (Effects on diet and habits)	−0.30, 0.015	−0.20, 0.123
Part 5 (Communication)	−0.35, 0.004	−0.32, 0.013
Part 6 (Concerns)	−0.27, 0.034	−0.23, 0.077
Part 7 (Emotional well-being)	−0.27, 0.035	−0.15, 0.271
Part 8 (Social life)	−0.24, 0.061	−0.26, 0.046
Part 9 (Physical symptoms)	−0.20, 0.112	−0.02, 0.901
Total	−0.35, 0.005	−0.28, 0.031
**SWAL-CARE** (Clinician’s assessment)	−0.05, 0.722	−0.10, 0.441

**Table 7:** Intercorrelation of quality-of-life questionnaires DHI and EAT-10.

DHI	Correlation Coefficient and *p*-Value before Treatment	Correlation Coefficient and *p*-Value after Treatment
Physical	0.669, <0.001	0.667, <0.001
Functional	0.737, <0.001	0.705, <0.001
Emotional	0.646, <0.001	0.675, <0.001

**Table 8:** Intercorrelation of quality-of-life questionnaires SWAL-QoL/SWAL-CARE and EAT-10.

Questionnaires	Correlation Coefficient and *p*-Value before Treatment	Correlation Coefficient and *p*-Value after Treatment
**SWAL-QoL**		
Part 1 (General statements)	−0.689, <0.001	0.716, <0.001
Part 2 (Eating aspects)	−0.583, <0.001	0.670, <0.001
Part 3 (Physical problems)	−0.663, <0.001	0.618, <0.001
Part 4 (Effects on diet and habits)	−0.487, <0.001	0.546, <0.001
Part 5 (Communication)	−0.554, <0.001	0.516, <0.001
Part 6 (Concerns)	−0.666, <0.001	0.649, <0.001
Part 7 (Emotional well-being)	−0.698, <0.001	0.661, <0.001
Part 8 (Social life)	−0.657, <0.001	0.522, <0.001
Part 9 (Physical symptoms)	−0.544, <0.001	0.377, 0.003
Total	−0.784, <0.001	0.736, <0.001
**SWAL-CARE** (Clinician’s assessment)	0.116, 0.360	0.279, 0.028

**Table 9:** Correlation of quality-of-life questionnaires DHI and SWAL-QoL/SWAL-CARE before and after complex OD treatment.

SWAL-QoL	Correlation Coefficient and *p*-Value before Treatment			Correlation Coefficient and *p*-Value after Treatment		
	DHI Physical, *p*-Value	DHI Functional, *p*-Value	DHI Emotional, *p*-Value	DHI Physical, *p*-Value	DHI Functional, *p*-Value	DHI Emotional, *p*-Value
Part 1 (General statements)	−0.703, <0.001	−0.738, <0.001	−0.614, <0.001	−0.574, <0.001	−0.559, <0.001	−0.540, <0.001
Part 2 (Eating aspects)	−0.650, <0.001	−0.688, <0.001	−0.705, <0.001	−0.686, <0.001	−0.654, <0.001	−0.602, <0.001
Part 3 (Physical problems)	−0.696, <0.001	−0.616, <0.001	−0.642, <0.001	−0.711, <0.001	−0.707, <0.001	−0.669, <0.001
Part 4 (Effects on diet and habits)	−0.620, <0.001	−0.665, <0.001	−0.531, <0.001	−0.625, <0.001	−0.583, <0.001	−0.609, <0.001
Part 5 (Communication)	−0.517, <0.001	−0.674, <0.001	−0.544, <0.001	−0.551, <0.001	−0.549, <0.001	0.597, <0.001
Part 6 (Concerns)	−0.651, <0.001	−0.707, <0.001	−0.705, <0.001	−0.596, <0.001	−0.641, <0.001	−0.638, <0.001
Part 7 (Emotional well-being)	−0.619, <0.001	−0.733, <0.001	−0.731, <0.001	−0.660, <0.001	−0.694, <0.001	−0.745, <0.001
Part 8 (Social life)	−0.678, <0.001	−0.738, <0.001	−0.703, <0.001	−0.609, <0.001	−0.656, <0.001	−0.634, <0.001
Part 9 (Physical symptoms)	−0.468, <0.001	−0.374, 0.002	−0.466, <0.001	−0.393, 0.002	−0.324, 0.010	−0.392, 0.002
Total	−0.777, <0.001	−0.794, <0.001	−0.784, <0.001	−0.774, <0.001	−0.772, <0.001	−0.769, <0.001
**SWAL-CARE**	0.137, 0.281	0.134, 0.291	0.118, 0.352	0.173, 0.179	0.194, 0.130	0.183, 0.154

## Data Availability

All the data are available from the corresponding author upon reasonable request.
